# Short-Term Prediction of COVID-19 Using Novel Hybrid Ensemble Empirical Mode Decomposition and Error Trend Seasonal Model

**DOI:** 10.3389/fpubh.2022.922795

**Published:** 2022-07-29

**Authors:** Dost Muhammad Khan, Muhammad Ali, Nadeem Iqbal, Umair Khalil, Hassan M. Aljohani, Amirah Saeed Alharthi, Ahmed Z. Afify

**Affiliations:** ^1^Department of Statistics, Abdul Wali Khan University Mardan, Mardan, Pakistan; ^2^Department of Computer Science, Abdul Wali Khan University Mardan, Mardan, Pakistan; ^3^Division of Computer Science, Mathematics and Science, St John's University, New York, NY, United States; ^4^Department of Mathematics & Statistics, College of Science, Taif University, Taif, Saudi Arabia; ^5^Department of Statistics, Mathematics and Insurance, Benha University, Benha, Egypt

**Keywords:** prediction, COVID-19, ensemble empirical mode decomposition, augmented Dicky-Fuller test, ARIMA, error trend seasonal model

## Abstract

In this article, a new hybrid time series model is proposed to predict COVID-19 daily confirmed cases and deaths. Due to the variations and complexity in the data, it is very difficult to predict its future trajectory using linear time series or mathematical models. In this research article, a novel hybrid ensemble empirical mode decomposition and error trend seasonal (EEMD-ETS) model has been developed to forecast the COVID-19 pandemic. The proposed hybrid model decomposes the complex, nonlinear, and nonstationary data into different intrinsic mode functions (IMFs) from low to high frequencies, and a single monotone residue by applying EEMD. The stationarity of each IMF component is checked with the help of the augmented Dicky–Fuller (ADF) test and is then used to build up the EEMD-ETS model, and finally, future predictions have been obtained from the proposed hybrid model. For illustration purposes and to check the performance of the proposed model, four datasets of daily confirmed cases and deaths from COVID-19 in Italy, Germany, the United Kingdom (UK), and France have been used. Similarly, four different statistical metrics, i.e., root mean square error (RMSE), symmetric mean absolute parentage error (sMAPE), mean absolute error (MAE), and mean absolute percentage error (MAPE) have been used for a comparison of different time series models. It is evident from the results that the proposed hybrid EEMD-ETS model outperforms the other time series and machine learning models. Hence, it is worthy to be used as an effective model for the prediction of COVID-19.

## Introduction

There has been a growing recognition among data analysts and researchers to focus on the prediction of COVID-19 in different parts of the world. The COVID-19 pandemic can be traced back to a group of severe pneumonia cases identified in Wuhan, China, in December 2019 (1). The initial spread of this contagious virus has been linked to a living animal seafood marketplace in Wuhan, pointing to a zoonotic source of the pandemic. However, person-to-person transmission has driven rapid spread with cumulative numbers reaching 53,164,803 reported cases, and 1,300,576 deaths globally since the start of the pandemic until 14 November 2020 (2). The worst-hit countries are Italy, France, Germany, and United Kingdom (UK) which recorded approximately 5,084,645 reported cases and 151,380 cumulative deaths. Extraordinary measures have been taken by these countries to reduce the viral spread, specifically in the densely populated regions to reduce the chances that sick people might come into contact with healthy ones. In recent times, the prediction of the current pandemic of COVID-19 outbreak is a test for data experts as inadequate information is available on the initial growing curve, and the epidemiological properties of the virus to be fully elucidated. There has been a renewed interest in using time series models to predict the epidemics, namely, SARS, Ebola, influenza, and dengue (3–10). These studies have shown an increasing curiosity in applying time series models as valuable tools in estimating and predicting epidemics. Unlike the regression models that need one response and at least one explanatory variable, univariate time series models are data-driven and can be used for forecasting without any explanatory variables. Predicting the daily confirmed cases and deaths from COVID-19 is hard as compared to the cumulative confirmed cases and deaths. The reason is that the daily data follow a nonlinear and nonstationary pattern, and hence, most of the linear time series models cannot capture its nonstationary characteristics more precisely.

In recent years, the empirical mode decomposition (EMD) and its modified form known as ensemble empirical mode decomposition (EEMD) (11, 12) have emerged as an attractive method for complex signal analysis. Using this method, a complex signal can be partitioned into a limited number of intrinsic mode functions (IMFs), having simpler frequency mechanisms that lead to easy and precise forecasting. The EMD has been extensively applied in numerous areas, such as the investigation of the complex nonlinear sea wave data (13), earthquake data analysis, construction state monitoring (14), diagnosis of faults in the machines (15, 16), prediction of stock markets, exchange rates, and crude oil (17–20).

Commonly, two approaches have been used in the past, i.e., the first one is statistical, and the second one is referred to as a mathematical model for the prediction of different pandemics. Following are a few studies available in the literature that shows the importance of these models to forecast the spread of the pandemics.

The method of serial interval (SI) of the infection was used by Zhao et al. (21) to estimate the value of reproduction rate (R_0_). By implementing this method, the estimated value of R_0_ for COVID-19 is found to be 2.56 with a 95% confidence band of 249–2.53. Based on the estimated value of R_0_, the initial cases of COVID-19 in China followed an exponential growth. The unreported cases from 1 January 2020 to 15 January 2020, are 469 with a 95% prediction interval of 403–540. It is concluded from this study that the unreported cases probably happen during the first 2 weeks of January.

To find out the predicted reproduction number R_0_ of COVID-19, the author in Tang et al. (22) used a deterministic compartmental mathematical model with other variables, namely, the progression of the disease, epidemiological status of the individuals, and intervention measures. The method of likelihood has been used for estimation; the estimated control reproduction number was found to be 6.47 with a 95% prediction interval of 5.71 to 7.23. It is also concluded from this study that tracing, isolation, and quarantine can decrease the reproduction and transmission rate of COVID-19.

Three different artificial neural networks (ANN), i.e., multilayer perception (MLP), radial basis function (RBF), and time delay neural network (TDNN) have been compared with the ARIMA model for predicting the hepatitis A virus (HAV) (23). They used 13 years of data on the HAV in Turkey to check the accuracy of ANN and ARIMA models. Based on the smallest values of mean squared error (MSE), normalized mean squared error (NMSE), and mean absolute error (MAE), the method of MLP outperforms other methods to forecast the infections caused by HAV.

A simple mean-field and susceptible-infected-recovered-death (SIRD) model was used by Fanelli et al. (24) to predict the dynamics of the COVID-19 in China, Italy, and France. The simple mean-field model can be used efficiently to find out the time and height of the peak of the cumulative confirmed cases. The peak of the COVID-19 in Italy is around 21 March 2020, with maximum cumulative cases of 2,600. Using the same data for the SIRD model, it is estimated that the recovery rate for the three different countries is the same, whereas rate of death and infection is different.

Different phenomenological models, i.e., the generalized logistic growth model (GLM), Richard growth model, and subepidemic growth models are implemented for the short-term real-time forecast of COVID-19 in the Hubei Province where the virus has been originated for the overall trajectory in China. Among different phenomenological models, the GLM and Richard model yield comparable prediction intervals in Hubei, while the subepidemic model gives a wider interval than the competing models. Furthermore, the prediction intervals obtained by the subepidemic model are much wider than the other two models both in Hubei and other provinces of China (25).

An exponential model has been used to forecast the number of infected people from COVID-19 in Italy (26). Based on the exponent value of *r*=*0.225* for the model, the exponential prediction and the actual number of confirmed cases are very much similar. According to this model, the estimated reproduction rate R_0_ varies between 2.76 and 3.25, which is very much similar to the one reported initially for the city of Wuhan in China. It is predicted from this model that the cumulative number of confirmed cases in Italy by March 15 will be more than 30,000.

The SIRD model was fitted by Anastassopoulou et al. (27) to estimate the basic reproduction number *R*_0_, daily confirmed cases, and daily deaths along with a 90% confidence interval. Based on the data of the confirmed cases, the average estimated and simulated value of R_0_ for the SIRD model is approximately 2.6 and 2. According to this study, the total number of infected people could reach 180,000 with a lower confidence interval of 45,000, and the total number of deceased persons from COVID-19 might be more than 2,700 by February 29. It is also evident from this study that the fatality rates show a declining pattern from January 26.

A simple time series predicting method from the exponential family to forecast the total number of infected people from COVID-19 was used by Petropoulos et al. (28). The forecast accuracy of this method is better than the other time series models and is hence used for short-term forecasting. Models from the exponential family capture both trends and seasonal components based on the nature of the data only trend, and nonseasonal components of the dataset are used in this study. The 10 days ahead forecasted value of cumulative confirmed cases around the globe is 209,000 with a 90% prediction interval from 38,000 to 534,000 in the time window from 01 February 2020 to 10 February 2020. Similarly, the last 10 days (from 12 March 2020 to 21 March 2020) ahead forecast of cumulative confirmed cases from COVID-19 in the entire world are 210,000.

The well-known ARIMA model was used by Benvenuto et al. (29) to predict the trend of the spread and prevalence of novel coronavirus. Autocorrelation function (ACF) and partial autocorrelation function (PACF) were used to estimate the parameters of the model. Based on the estimated values of the parameters, ARIMA (1,0,4) and ARIMA (1,0,3) were used to predict the prevalence and incidence of the COVID-19. The forecasted values based on the two ARIMA models of prevalence and incidence for the time window from 11 February 2020 to 02 February 2020 (2 days) are 45,151 and 2,418 with prediction intervals of (42,084 and 48,218) and (1,534 and 3,302), respectively.

An improved adaptive neuro-fuzzy inference system (ANFIS) based on an enhanced flower pollination algorithm (FPA) and slap swarm algorithm (SSA) was proposed by Al-Qaness et al. (30) to forecast the 10 days of cumulative confirmed cases from COVID-18 in China. The performance of the model has been increased by determining the parameters of both the ANFIS and FPASSA models. The efficiency of the proposed method in terms of RMSE, MAE, and MAPE is better than the other models. Based on the FPASSA-ANFS model, the estimated number of cumulative confirmed cases by 28 February 2020, in China is 99,453.

The well-known univariate time series ARIMA model was used to predict the cumulative confirmed cases, deaths, and recoveries from COVID-19 in Pakistan. Based on the investigational results of this study, ARIMA (0, 2, 1) (1, 0, 0) outperformed other time series models for predicting the next 10 days' cumulative confirmed cases. Similarly, ARIMA (0,2,1) was found to be the best candidate model for forecasting aggregate recoveries and deaths (31).

The problem of predicting the daily confirmed and daily deaths from COVID-19 has gained limited attention in the literature. Although some attempts have been made to address this issue, it is still a potential area to be investigated. Literature offers no clear methodology for the problem of predicting the daily confirmed and deaths from COVID-19. Here, we report a neglected aspect in previous studies, and an attempt has been made to address the issue with a more sophisticated and simple hybrid model. It can be observed from the graphical representation of the daily confirmed cases and deaths, as shown in **Figures 2**, **3**, which follow a nonlinear and nonstationary pattern that cannot be predicted easily by using any linear statistical or mathematical models.

To capitalize the strength of these models and address the issues and weaknesses of the abovementioned models, an attempt has been made to predict the daily confirmed cases and deaths from COVID-19 by suggesting a new hybrid EEMD-ETS model whose detailed description is outlined in the “Proposed hybrid EEMD-ETS model” section.

Since the daily confirmed cases and deaths from COVID-19 follow an irregular pattern, therefore, the traditional time series models might not enhance their nonlinear and stochastic characteristic and thus produce very unrealistic prediction results. This has been achieved primarily through the use of EEMD. The first step of this method is to decompose the nonlinear pattern of the data into dissimilar IMFs, and a single monotone residue component followed by the selected IMFs is then used to build the hybrid ETS model, which is then used for short-term prediction.

The novelty in this article is the development of a hybrid time series model which is based on the well-known idea of a divide-and-conquer algorithm that works recursively by breaking down the nonlinear COVID data into subgroups technically known as IMFs and then efficiently predicts COVID-19 in Italy, Germany, UK, and France.

The remaining article is organized in the following sections with techniques for future predictions in the “Prediction methods” section and the proposed hybrid EEMD-ETS model in the “Proposed hybrid EEMD-ETS model” section; experimental results on four COVID-19 datasets of Italy, France, Germany, and the UK are briefly explained in the “Experimental results” section, followed by discussion, and finally the conclusion is presented.

## Prediction Methods

In this section, we provided the details of the experimental procedures carried out in this study. Numerous research articles have shown that the time series forecasting model's emphasis on the past behavior of a random phenomenon best captures the underlying trends and patterns. The ideal model is then employed for the prediction of the future behavior of the underlying study variable. Over the past few years, there have been fabulous efforts carried out on the expansion of different time series models for forecasting the spread of contagions. In this article, we have suggested a hybrid technique that is based on EEMD and error trend seasonality (ETS) to predict the daily confirmed cases and deaths from COVID-19. A brief explanation of all the time series methods is outlined along with the proposed method in the following subsections.

### Mean Method

In this method of forecasting, the mean value of all the historical time series is equal to the future forecast value. If we denote the historical time series values by *x*_1_*, x*_2_*, ..., x*_*t*_, then the future forecast value of *the k* period ahead is given by


(1) $${\hat{x}}_{t+k}=\;\underline{\text{x}}=\left(x_{1}+x_{2}+x_{3}+\ldots +x_{t}\right)/t$$

### Simple Exponential Smoothing

The simple exponential smoothing (SES) technique is one of the most common techniques of exponentially smoothing methods. Consider a time series *x*_1_*, x*_2_*, ..., x*_*t*_ with no seasonal or symmetric trend, the future forecasted value ${\hat{x}}_{t+k}$ is a weighted sum of the past values


(2) $$\begin{array}{r} {\hat{x}}_{t+k}=a_{0}x_{t}+a_{1}x_{t-1}+a_{2}x_{t-2}+\ldots \end{array}$$

where *{a*_*i*_*}* are weights in such a manner that more weights are given to the most recent values and fewer weights to the values that lie far away in the past. When the weights are increasing geometrically, the final equation for SES becomes


(3) $${\hat{x}}_{t+k}=\gamma x_{t}+\gamma \left(1-\gamma \right)x_{t-1}+\gamma {\left(1-\gamma \right)}^{2}x_{t-2}+\ldots$$

### Naïve Method

This method of forecasting works very efficiently for many economic and financial time series, especially when the time series follows random walks, that is why this method is sometimes known as the random walk forecasting method. In this method of point forecasting, the future forecast value is equal to the value of the last observation, i.e.,


(4) $$\begin{array}{r} {\hat{x}}_{t+k}=x_{t} \end{array}$$

### Theta Model

This theta model was proposed by Assimakopoulos and Nikolopoulos (32), where the basic idea of this forecasting method is altering the local curvature of the univariate time series through a coefficient known as “Theta” (θ) which is directly applied to the second difference of the time series. Therefore, a new series of time series known as Theta-lines are constructed and denoted as *L*(θ). Each of these Theta-lines is extrapolated individually and the forecasts are aggregated either equally weighted or through a weighed optimization procedure. Consider that the initial time series *Y* = [*Y*_1_, *Y*_2_, *Y*_3_, …, *Y*_*t*_] is decomposed into two Theta-lines, i.e., *L*(θ = 0) *and L*(θ = 2), then the algebraic equation for the model in its modified form is as follows:


(5) $$\begin{array}{r} Y_{t}=\frac{1}{2}\left(L_{t}\left(\theta =0\right)+L_{t}\left(\theta =2\right)\right),\;\forall \;t=1,2,\ldots ,n \end{array}$$

### TBATS Model

The trigonometric seasonality Box–Cox transformation ARIMA errors trend seasonal (TBATS) model developed by De Livera et al. (33) uses a combination of Fourier terms with an exponential smoothing state-space model and a Box–Cox transformation in an entirely automatic method. There is a slight difference between harmonic regression and the TBATS model, in the sense that the seasonal patterns are repeated without changing for the time in harmonic regression, while in the TBATS model, the seasonal components change slowly over time. The matrices for the TBATS model can be written as = (1, ϕ, *a*, φ, θ)′ , $g={\left(\alpha ,\beta ,\gamma ,1,0_{p-1},1,0_{q-1}\right)}^{\prime }$, and


(6) $$F=\left[\begin{array}{lllll} 1 & \phi & 0_{\tau } & \text{α}\phi & \text{α}\theta \\ 0 & \phi & 0_{\tau } & \text{β}\phi & \text{β}\theta \\ {0^{\prime }}_{\tau } & {0^{\prime }}_{\tau } & A & B & C\\ 0 & 0 & 0_{\tau } & \varphi & \theta \\ {0^{\prime }}_{p-1} & {0^{\prime }}_{p-1} & O_{p-1,t} & I_{p-1,p} & O_{p-1,q}\\ 0 & 0 & 0_{\tau } & 0_{p} & 0_{q}\\ {0^{\prime }}_{q-1} & {0^{\prime }}_{q-1} & O_{q-1,\tau } & O_{q-1.p} & I_{q-1,q} \end{array}\right]$$

Here, if all the components in the TBATS model are available, then these matrices are valid but if any of the components of the model is not available, then the corresponding term must be omitted from the matrices too.

### The Holt-Winters Linear Trend Forecasting Procedure

This method of forecasting is the generalization of the SES technique by introducing two smoothing parameters α, γ for updating the local level *(L*_*t*_*)* and trend *(T*_*t*_*)* components of the time series. The values of these smoothing parameters generally fall in the range of (0, 1). The one forecast and two smoothing equations for the level and trend are given by


(7) $$\begin{array}{r} {\hat{x}}_{t+k}=L_{t}+kT_{t} \end{array}$$

(8) $$\begin{array}{r} L_{t}=\alpha x_{t}+\left(1-\alpha \right)\left(L_{t-1}+T_{t-1}\right) \end{array}$$

(9) $$\begin{array}{r} T_{t}=\gamma \left(L_{t}-L_{t-1}\right)+\left(1-\gamma \right)T_{t-1} \end{array}$$

It can be seen from the level component given in equation 7 that *L*_*t*_ is a weighted average of the observations *x*_*t*_ and the one-step-ahead forecast given by (*L*_*t*−1_+*T*_*t*−1_). Similarly, the trend component given in equation 8 indicates that *T*_*t*_ is the weighted average of the estimated trend at time t based on (*L*_*t*_−*L*_*t*−1_) and *L*_*t*−1_. The final *k*-step-ahead forecasted values are the linear combination of the last estimated level *L*_*t*_ and *k* times the last estimated trend values *T*_*t*_.

### Damped Trend Methods

The motivation behind this forecasting technique is the limitation of Holt's linear trend method that exhibits an endless trend component in the future horizon either increasing or decreasing those results in over-forecast, specifically for longer horizons. To overcome this drawback, Gardner and McKenzie (34) introduced a parameter that dampens the effect of the trend component in the future, and the values of this dampen parameter also lie in the range (0, 1). Mathematically, the holt-linear method is modified by incorporating the dampen parameter, i.e.,


(10) $$\begin{array}{r} {\hat{x}}_{t+k}=L_{t}+\left(\psi +\psi^{2}+\psi^{3}+\ldots +\psi^{k}\right)T_{t} \end{array}$$

(11) $$\begin{array}{r} L_{t}=\alpha x_{t}+\left(1-\alpha \right)\left(L_{t-1}+\psi T_{t-1}\right) \end{array}$$

(12) $$\begin{array}{r} T_{t}=\gamma \left(L_{t}-L_{t-1}\right)+\left(1-\gamma \right)\psi T_{t-1} \end{array}$$

### ARIMA

This technique was first introduced by Box and Jenkins (35) and has been widely used for univariate time series forecasting. This method is completely data-driven, with the forecasted values of a variable depending upon the past or lagged values of the same variable. In terms of *Y*_*t*_, the general forecasting equation is


(13) $$\begin{array}{rcl} Y_{t} & = & \beta +\alpha_{1}Y_{t-1}+\alpha_{2}Y_{t-2}+\ldots +\alpha_{p}Y_{t-p}+\varepsilon_{t}-\varphi_{1}\varepsilon_{t-1}\\ \;-\varphi_{2}\varepsilon_{t-2}-\ldots -\varphi_{q}\varepsilon_{t-q} \end{array}$$

Here, the moving average parameters φ′*s* are described so that their signs are negative in the equation, following the convention presented by Box and Jenkins. Several researchers and software (i.e., the R) described them so that they have plus signs as an alternative. When real values are plugged into the equation, there is no doubt, but it is significant to distinguish which rules the software practices when interpreting the output. Often, the parameters are denoted by *AR (1), AR (2),…* and *MA (1), MA (2)*, …. To recognize a suitable ARIMA model for *Y*_*t*_, starting from the order of differencing *(d)* demanding to make the series stationary and eliminate the unstructured characteristics of seasonality, possibly in combination with a variance-stabilizing conversion, such as logging or deflating. If you end at this point and predict that the differenced series is constant, you have merely fitted a random walk or random trend model. However, the stationary series may still have auto-correlated errors, signifying that some values of *AR* terms *(p* ≥ *1)* and/or some number *of MA* terms *(q* ≥ *1)* are also desirable in the forecasting equation. The procedure of determining the values of *p, d*, and *q* that are excellent for a specified time series and plots of ACFs and partial autocorrelation functions (PACFs) will be used for this purpose.

### Neural Network Autoregression

**Figure 1:**
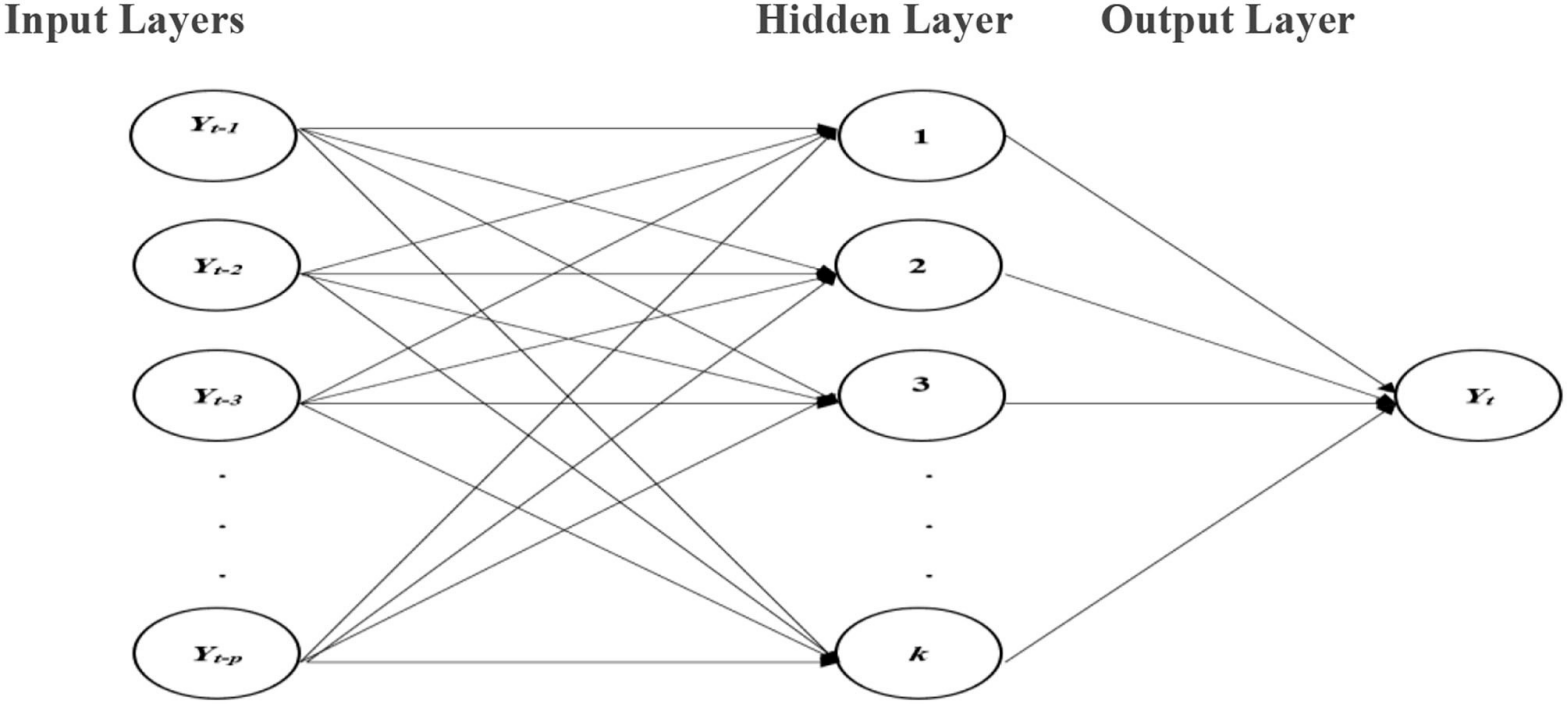
NNAR model with *p* autoregressive terms as inputs and one hidden layer with *k* nodes.

The ANNs are forecasting techniques that are founded on easy mathematical models of the brain. They permit compound nonlinear associations amid the response and predictor variables. A neural network is similar to a network of “neurons” which are ordered in layers. The predictors (or inputs) form the bottom layer, and the forecasts (or outputs) form the top layer. In the case of time series data, the lagged values can be used as inputs to the neural network and are known as the neural network autoregression (*NNAR*) model. In this study, we consider only the feed-forward neural network with one hidden layer denoted by *NNAR (p, k)*, meaning that there are *p* lagged inputs and *k* nodes in the hidden layer. A schematic diagram of the *NNAR* model is shown in Figure 1.

### Long Short-Term Memory Model

Long short-term memory (LSTM) models belong to the artificial recurrent neural network (RNN) architecture that is widely used to handle sequence dependence in complex problem domains, namely, machine learning translations, speech recognition, handwriting recognition, and anomaly detection in network traffic of IDSs (intrusion detection systems). LSTM networks are compatible to classify, process, and make forecasts based on time series data with different activation functions, namely, sigmoid, hyperbolic, and hyperbolic tangents. LSTMs were established to solve the problem of vanishing gradients and exploding gradients that can be confronted by the traditional RNNs during the training phase (36). The most common LSTM network is comprised of a cell, an input gate, an output gate, and a forget gate. The cell recalls values over arbitrary time intervals, and the three gates control the flow of data into and out of the cell. The mathematical equations for the forward pass of an LSTM unit with forget gate are


(14) $$\begin{array}{r} m_{t}=\vartheta_{g}\left(R_{m}y_{t}+P_{m}l_{t-1}+a_{m}\right) \end{array}$$

(15) $$\begin{array}{r} n_{t}=\vartheta_{g}\left(R_{n}y_{t}+P_{n}l_{t-1}+a_{n}\right) \end{array}$$

(16) $$\begin{array}{r} o_{t}=\vartheta_{g}\left(R_{o}y_{t}+P_{o}l_{t-1}+a_{o}\right) \end{array}$$

(17) $$\begin{array}{r} {\tilde{d}}_{t}=\vartheta_{g}\left(R_{d}y_{t}+P_{d}l_{t-1}+a_{d}\right) \end{array}$$

(18) $$d_{t}=m_{t}{}^{\circ}d_{t-1}+n_{t}{}^{\circ}{\tilde{d}}_{t})$$

(19) $$\begin{array}{r} l_{t}=o_{t}∙\vartheta_{l}\left(c_{t}\right) \end{array}$$

where the initial values of *d*_0_ and *l*_0_ are both equal to zero, and the operator ° is identified as a Hadamard product.

### Error Trend and Seasonal Model

This method can forecast trends and seasonal components and is thus suitable for predicting the univariate time series. The ETS model is a special case of exponential smoothing models known as state-space models. There are different versions of these models which can be represented by the error, trend, and seasonality types, generally, a three-character string classifying method. The error component is denoted by the first letter (“A,” “M,” or “Z”); the trend type is represented by the second letter (“N,” “A,” “M,” or “Z”); and the season type is represented by the third letter (“N,” “A,” “M,” or “Z”). In all of these scenarios, “N” = none, “A” = additive, “M” = multiplicative, and “Z” = automatic selection. Therefore, for example, the SES with additive errors is denoted by “ANN”; similarly, the multiplicative Holt-Winters' method with multiplicative errors is “MAM” and so forth.

There are 30 models with different combinations of error, trend, and seasonality (37). Supplementary Table 1 shows different combinations of these models. These models have fitted automatically to the data by using the method of maximum likelihood (ML) by optimizing the smoothing parameters and initial conditions with the help of a simple optimizer (38).

where N, M, A, A_d_, and M_d_ denote None, Multiplicative, Additive, Damped Additive, and Damped multiplicative, respectively. Akaike's information criteria (AIC) and Bayesian information criteria (BIC) will be used for the selection of the best candidate ETS model. An ETS model with a minimum AIC value among the considered models will be chosen for application. The mathematical structure of AIC and BIC is given below (39, 40).


(20) $$\begin{array}{r} AIC=-2^{*}\ln\ln\left(l\right)+2^{*}p \end{array}$$

(21) $$\begin{array}{r} BIC=-2^{*}\ln\ln\left(l\right)+2^{*}\ln\ln{\left(n\right)}^{*}p \end{array}$$

### Ensemble Empirical Mode Decomposition

The method of EMD uses the well-known Hilbert–Huang transform (HHT) technique to decompose the complex signal into dissimilar oscillatory components varying from low to high frequency and a single monotone residue (7). These oscillatory functions are technically known as IMFs. There are two basic conditions for each IMF: (i) the difference between the number of extrema and the number of zero-crossing will be one, and (ii) the upper and lower envelope will have zero mean. Given a signal *y(t)*, the algorithm of EMD can be used successfully to divide signals into their different components (11, 12, 41, 42). This method is robust, simple, and efficient that does not require any strong model assumptions. It is worthy to mention that some authors have used this method for the prediction of different complex and nonlinear time series datasets (43–45). The issue with this method relates to the problem of mode mixing which refers to the situation when an IMF resulting from EMD decomposition has components of different frequencies. Numerous efforts have been made on solving the problem of mode mixing and thus EEMD is one such alternative approach (8). This method has the flexibility to handle very complex signals without the mode mixing problem. In this technique, the white noise would be added to fill in the whole time–frequency space homogeneously, which can smooth an accepted separation of the frequency scales and diminish the existence of mode mixing. According to the properties of the EMD method, the procedure of EEMD can be described as follows:

Step 1: Add a random Gaussian white noise *n*_*i*_*(t)* to the original time series *y(t)*, the noise-added signal *y*_*i*_*(t)* is as follows:
  (22) $$\begin{array}{r} y_{i}\left(t\right)=y\left(t\right)+n_{i}\left(t\right) \end{array}$$
Step 2: Recognize all the local extrema (local maxima and minima) in the new signal *{y*_*i*_ *(t)}*.
Step 3: Find out the upper *{U (t)}*, and lower envelope *{L (t)}* in the new white noise added signal *y*_*i*_*(t)*.
Step 4: Join all the local extrema through the cubic spline interpolation technique to find out the mean of both the upper and lower envelope, i.e., *M (t)*:
  (23) $$\begin{array}{r} Mean\left(t\right)=\frac{U\left(t\right)+L\left(t\right)}{2} \end{array}$$
Step 5: The mean envelope calculated in step 4 will be subtracted from the actual signal to obtain the first component, i.e.,
  (24) $$\begin{array}{r} k_{1}\left(t\right)=y\left(t\right)-Mean\left(t\right) \end{array}$$
  If *k*_1_ *(t)* meets the two properties of the IMF defined above, then it should be well-thought-out as the first IMF; else, steps 1 to 5 will be repeated by considering *k*_1_ *(t)* as a new-fangled signal.
Step 6: The first IMF obtained in step 5 will be deducted from the signal *y (t)* to obtain *r*_1_ *(t)*, i.e.,
  (25) $$\begin{array}{r} r_{1}\left(t\right)=y\left(t\right)-k_{1}\left(t\right)\ldots \left(25\right)\; \end{array}$$
Step 7: In this step, *r*_1_ *(t)* will be considered as a new signal and the sifting process of step 1 will be applied once again. The above process will continue until the last IMF is taken out from the signal. The overall trend of the signal will be a smooth monotonic residue obtained in the last step of EMD, and finally, the actual signal *y (t)* can be decomposed as:
  (26) $$\begin{array}{r} y\left(t\right)=\overset{n}{\underset{i=1}{\sum }}k_{i}\left(t\right)+r_{n}\ldots \left(26\right)\; \end{array}$$
  where *r*_*n*_ is the residue and *k*_1_*(t), k*_2_*(t),….,k*_*n*_*(t)* are different IMFs with different frequencies that vary from high to low. The final results of this decomposition are shown in Supplementary Figures 1–4.

It can be observed that the EEMD approach produces good quality results in terms of breaking the variations into their different components. In the first step, we decomposed the data into their different subparts varying from high- to low-frequency IMFs and a single monotone residual component. The results of this decomposition are presented and can be verified from Supplementary Figures 1–4 given earlier. For all the four countries, seven (07) different IMFs are obtained for both the daily confirmed cases and daily deaths. After rigorous examination, it is revealed from these IMFs that there are two types of variation in the COVID-19 data, i.e., short term and long term. There are different reasons for short-term fluctuations that bring ups and downs in the daily confirmed cases and daily deaths, namely, imposing new restrictions, building emergency hospitals, and facilitating patients in intensive care units (ICU). These IMFs justify that any linear, mathematical, or statistical model will not produce good forecasting results unless they are used on the cumulative number of confirmed cases and the number of deaths.

## Proposed Hybrid EEMD-ETS Model

**Figure 2:**
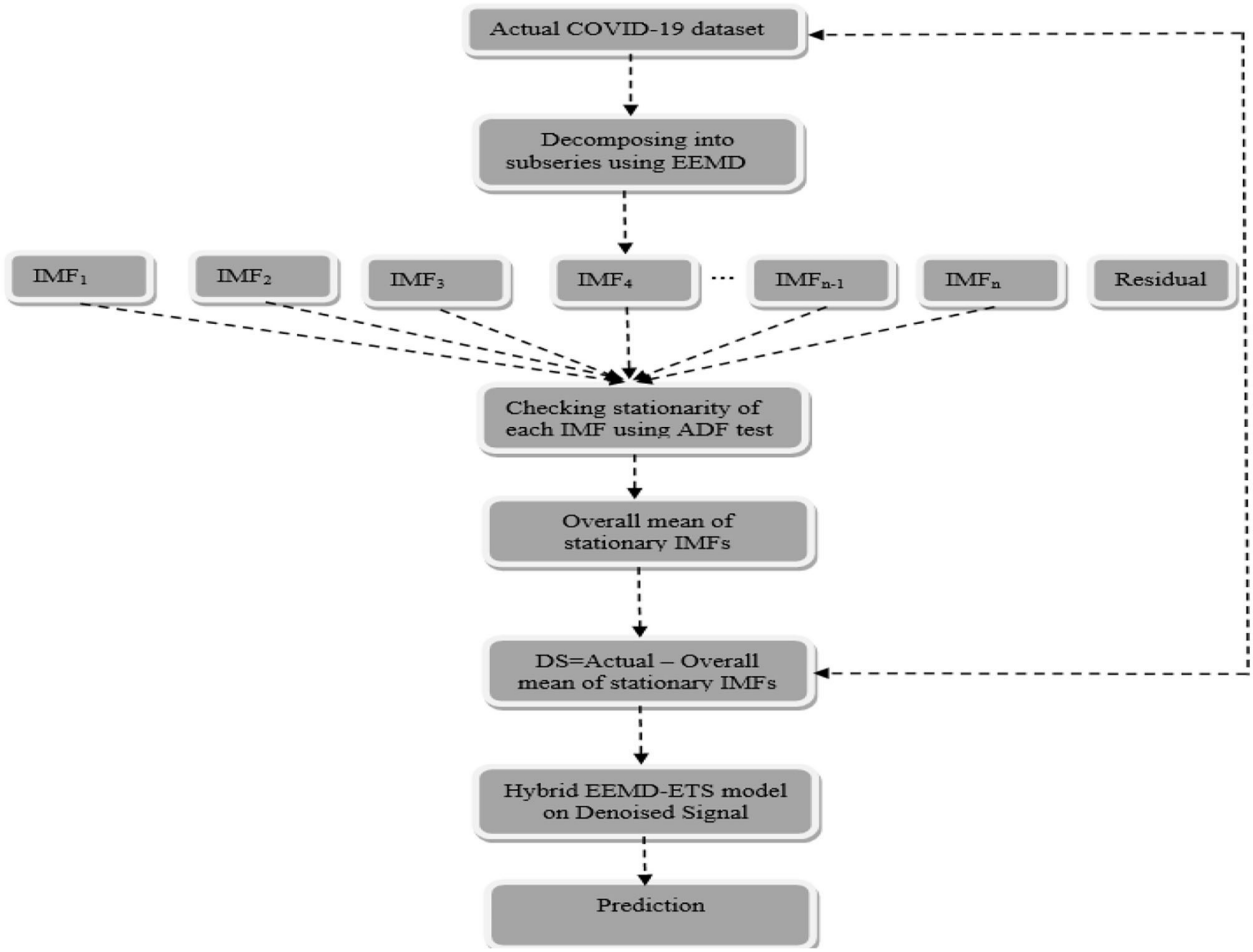
Flowchart of the proposed hybrid EEMD-ETS model. Where “DS” means denoised signal.

The idea behind the proposed model is based on the well-famous divide-and-conquer algorithm that decomposes a given problem into multiple subproblems and their results are then combined efficiently. The proposed idea can be seen as a two-stage process, and the method of EEMD is implemented to decompose the nonlinear and nonstationary COVID-19 time series data into different IMFs in the first place and then the proposed method belongs to building the novel hybrid model in the second stage. The whole procedure is schematically shown in Figure 2, followed by a step-by-step implementation of the proposed hybrid model.

**Step 1**. The method of EEMD define above is used to decompose the actual COVID-19 daily confirmed cases and deaths data of all the four countries into different IMFs and residues.
**Step 2**. After decomposing the daily confirmed cases and deaths data into different IMFs and monotone residue in step 1, the proposed hybrid model is developed based on univariate time series ETS that belongs to the exponential family.
**Step 3**. In this step, the stationarity of each IMF is checked with the help of the augmented Dickey–Fuller (ADF) test (36). The ADF test is a well-known technique to test the null hypothesis that a unit root is present in the time series data. The alternative hypothesis is usually considered that the under-observation time series data are stationary. The results of this test are presented in Supplementary Table 3, Tables 1–4. After dividing IMFs into a non-overlapping sequence of stationary and nonstationary components, the overall mean of the stationary IMFs is subtracted from the actual data, to get the denoising signal, i.e.,
  (27) $$y_{N}\left(t\right)=x\left(t\right)-G.Mean[St(IMF\left(t\right)]$$
  where *y*_*N*_*(t)* is the new denoised univariate time series data, *x(t)* is the original data, and *G. Mean [SIMF(t)]* is the overall mean of the stationary IMFs.
**Step 4**. The univariate time series denoised signal is given as input to build the ETS model. The summary of each of these fitted ETS models is presented in Table 4, showing the corresponding values of smoothing parameters, the values of AIC and BIC, and the type of the best ETS model fitted.
**Step 5**. Once the ETS model is developed for the denoising data, the next step is to predict the future daily confirmed cases and deaths from COVID-19 for Italy, France, Germany, and the UK.
**Step 6**. Finally, the comparison is made between the predicted and hold-out datasets. Contrary to the traditional method of dividing the dataset into 80% training and 20% testing, the validity of this novel approach is demonstrated by using 259 observations out of 266 for model training, and the remaining 7 observations for checking its validity. Four statistical measures, i.e., root mean square error (RMSE), MAE, mean absolute percentage error (MAPE), and systematic mean absolute percentage error (sMAPE) (46) are used as a performance assessment criterion for the proposed model. The final results of these met1rics measures for the proposed and considered models are presented in Supplementary Table 4, Tables 5–7 for all four countries.

**Table 1:** ADF test results along with the overall mean for Italy.

**Component**	**ADF test value**	***P*-value**	**Decision**	**Mean**
IMF1_confirmedcases_	1.9403	0.99	Non-stationary	Not required
IMF1_dailydeaths_	−7.686	0.01	Stationary	−0.334
IMF2_confirmedcases_	−10.868	0.01	Stationary	−11.384
IMF2_dailydeaths_	−11.444	0.01	Stationary	0.102
IMF3_confirmedcases_	−6.2013	0.01	Stationary	26.675
IMF3_dailydeaths_	−3.922	0.0133	Stationary	−9.361
Overall Mean of daily confirmed cases	−9.49
Overall Mean of daily deaths	−3.197
IMF4_dailyconfirmed_	−2.95	0.175	Non-stationary	Not required
IMF4_dailydeaths_	−3.446	0.068	Non-stationary	Not required
IMF5_confirmed_	−1.249	0.891	Non-stationary	Not required
IMF5_deaths_	2.209	0.99	Non-stationary	Not required
IMF6_confirmed_	−1.5308	0.773	Non-stationary	Not required
IMF6_deaths_	2.84	0.99	Non-stationary	Not required
IMF7_confirmed_	−0.049	0.99	Non-stationary	Not required
IMF7_deaths_	1.578	0.99	Non-stationary	Not required

**Table 2:** ADF test results along with the overall mean for France.

**Component**	**ADF test value**	***P*-value**	**Decision**	**Mean**
IMF1_confirmedcases_	−9.0304	0.01	Stationary	75.483
IMF1_dailydeaths_	−7.842	0.01	Stationary	−6.631
IMF2_confirmedcases_	−8.943	0.01	Stationary	−37.356
IMF2_dailydeaths_	−7.006	0.01	Stationary	1.114
IMF3_confirmedcases_	−5.846	0.01	Stationary	−35.881
IMF3_dailydeaths_	−5.589	0.01	Stationary	5.192
IMF4_dailyconfirmed_	−4.132	0.01	Stationary	115.706
IMF4_dailydeaths_	−5.052	0.01	Stationary	−28.361
The overall mean of daily confirmed cases	29.488
The overall mean of daily deaths	−7.171
IMF5_confirmed_	−0.59	0.9773	Not stationary	Not required
IMF5_deaths_	1.067	0.99	Not stationary	Not required
IMF6_confirmed_	−2.2627	0.4652	Not stationary	Not required
IMF6_deaths_	2.243	0.99	Not stationary	Not required
IMF7_confirmed_	0.0833	0.99	Not stationary	Not required
IMF7_deaths_	−0.0373	0.99	Not stationary	Not required

**Table 3:** ADF test results along with the overall mean for Germany.

**Component**	**ADF test value**	***P*-value**	**Decision**	**Mean**
IMF1_confirmedcases_	−6.904	0.01	Stationary	20.086
IMF1_dailydeaths_	−7.607	0.01	Stationary	0.48
IMF2_confirmedcases_	−7.353	0.01	Stationary	−8.189
IMF2_dailydeaths_	−10.394	0.01	Stationary	0.2
IMF3_confirmedcases_	−3.607	0.032	Stationary	12.276
IMF3_dailydeaths_	−4.551	0.01	Stationary	0.619
IMF4_dailyconfirmed_	−3.579	0.0356	stationary	−133.804
IMF4_dailydeaths_	−3.396	0.055	Stationary	−7.759
Overall Mean of daily confirmed cases	−27.407
Overall Mean of daily deaths	−1.614
IMF5_confirmed_	−0.965	0.9427	Not stationary	Not required
IMF5_deaths_	1.078	0.99	Not stationary	Not required
IMF6_confirmed_	−1.466	0.8005	Not stationary	Not required
IMF6_deaths_	1.913	0.99	Not stationary	Not required
IMF7_confirmed_	0.022	0.99	Not stationary	Not required
IMF7_deaths_	−0.039	0.99	Not stationary	Not required

**Table 4:** ADF test results along with the overall mean for UK.

**Component**	**ADF test value**	***P*-value**	**Decision**	**Mean**
IMF1_confirmedcases_	−5.529	0.01	Stationary	−29.176
IMF1_dailydeaths_	−6.651	0.01	Stationary	−2.736
IMF2_confirmedcases_	−8.915	0.01	Stationary	−2.778
IMF2_dailydeaths_	−7.875	0.01	Stationary	0.385
IMF3_confirmedcases_	−4.912	0.01	Stationary	−21.64
IMF3_dailydeaths_	−4.523	0.01	Stationary	7.43
IMF4_dailyconfirmed_	−3.813	0.018	Stationary	−15.715
IMF4_dailydeaths_	−3.553	0.0381	Stationary	−24.06
The overall mean of daily confirmed cases	−17.33
The overall mean of daily deaths	−4.745
IMF5_confirmed_	−1.457	0.8044	Not stationary	Not required
IMF5_deaths_	1.51	0.99	Not stationary	Not required
IMF6_confirmed_	−3.838	0.99	Not stationary	Not required
IMF6_deaths_	1.1087	0.99	Not stationary	Not required
IMF7_confirmed_	0.69	0.99	Not stationary	Not required
IMF7_deaths_	0.1228	0.99	Not stationary	Not required

## Experimental Results

### Datasets

**Figure 3:**
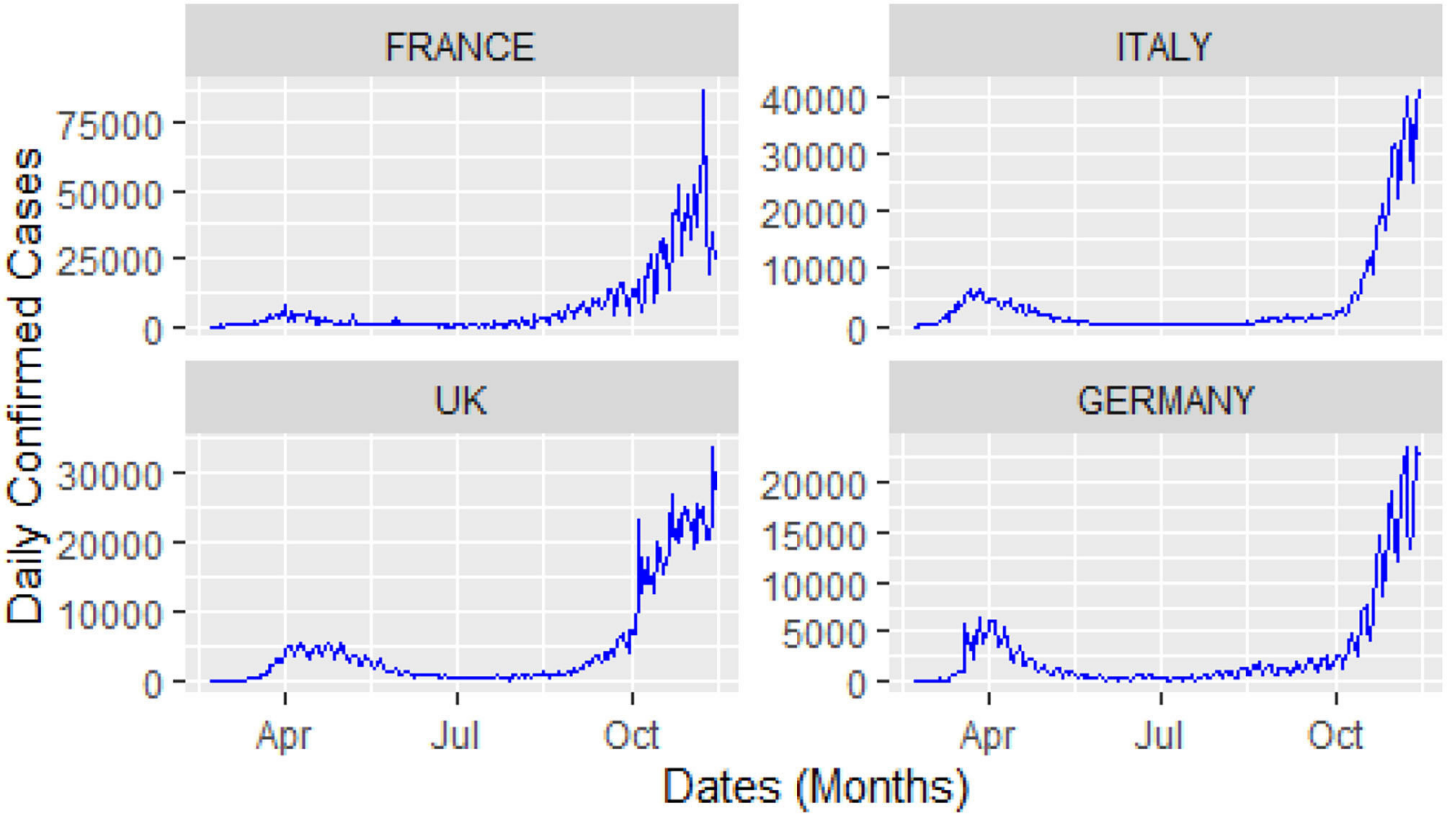
Daily confirmed cases: time window from 23 February 2020 to 14 November 2020.

**Figure 4:**
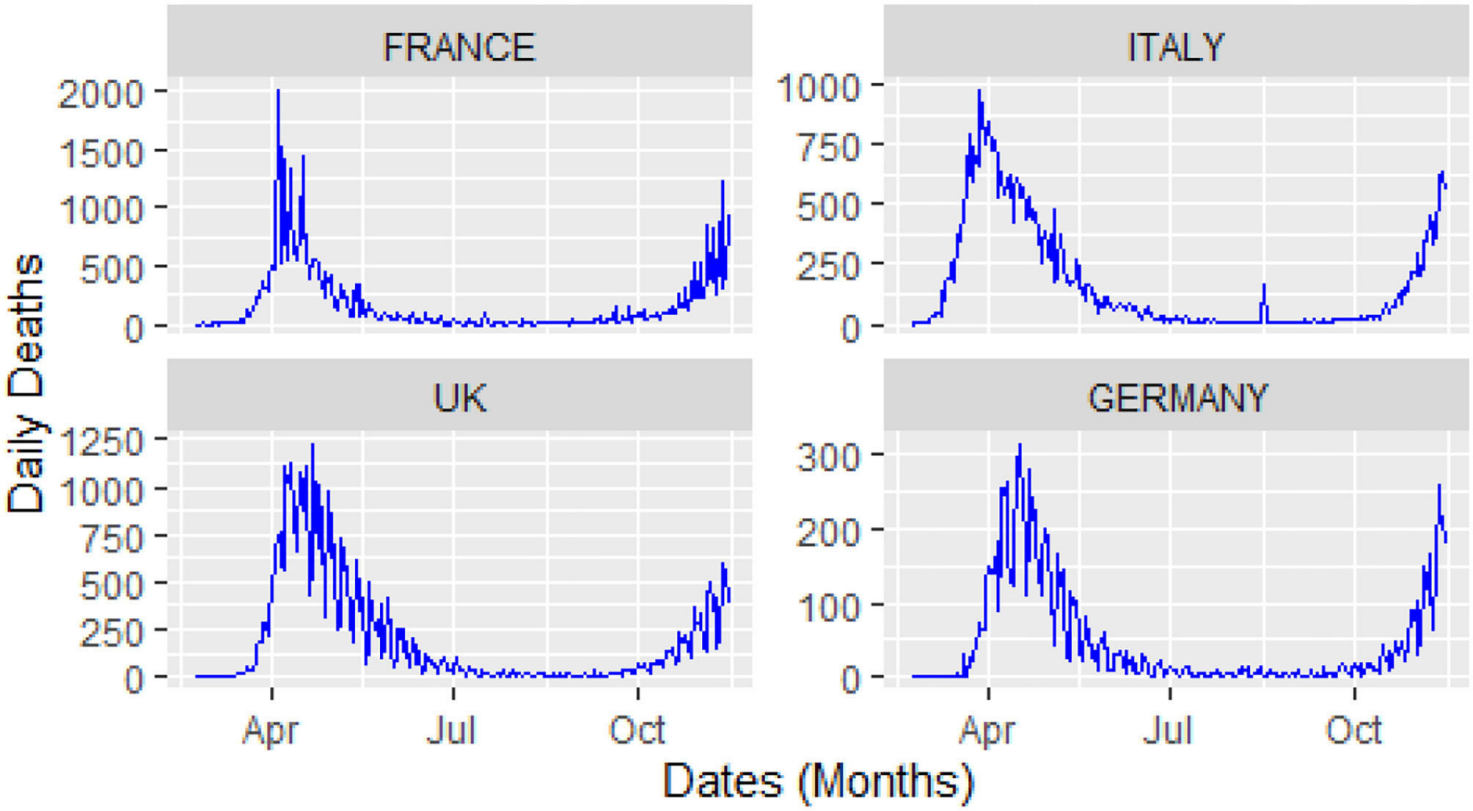
Daily deaths: time window from 23 February 2020 to 14 November 2020.

For this study, COVID-19 time series data on the number of daily confirmed cases, and the number of daily deaths were collected for four major European countries, i.e., Italy, United Kingdom (UK), Germany, and France from the website of the World Health Organization (WHO) during 23 February 2020, and 14 November 2020. A visual representation of the confirmed cases and deaths data for these countries is shown in Figures 3, 4.

From Figure 4, it can be observed that the curves of the deaths flatten after attaining peak value, followed by a second spike that occurs in august after the relaxation in the restrictions across Europe. Keeping in view of the trajectory of COVID-19, these four countries implemented a second lockdown to stop the further spread of the virus and save the precious lives of their citizens.

The analysis of these figures suggests the nonlinear and nonlinear pattern of COVID-19 daily data for the number of confirmed cases and the number of deaths; therefore, it cannot be predicted more accurately with any linear time series or mathematical models. Thus, based on the nature of the data, a more robust technique is required to accurately predict the COVID-19 in these four countries.

An overall descriptive summary for the study variables is given in Supplementary Tables 2, 3, confirming that Italy, the UK, Germany, and France are the most affected countries by COVID-19 in Europe with more than 5 million cumulative confirmed cases and 151,380 cumulative deaths. Based on these statistics, it implies that Germany has taken all the protective measures issued by WHO to stop the spread of COVID-19 with only 2,908 average daily cases. Similarly, their health system efficiently managed the hospitalized patients which seems to be the only reasonable reason that is why the average of daily and cumulative deaths in Germany are slowed as compared to their neighboring countries. Similarly, the standard deviation (SD) of the daily confirmed cases and deaths is minimum for Germany, indicating that the data points tend to be very close to the average which showed their resilience against the contagious COVID-19.

### Forecast Accuracy Criteria

The performance of the proposed approach and its resilience can be assessed by the following four statistical measures. The mathematical expressions of these four-performance metrics are given as follows:


(28) $$\begin{array}{r} RMSE=\sqrt{\frac{1}{N}\overset{N}{\underset{t=1}{\sum }}{\left(A_{t}-P_{h,t}\right)}^{2}} \end{array}$$

(29) $$\begin{array}{r} MAE=\frac{1}{N}\overset{N}{\underset{t=1}{\sum }}|A_{t}-P_{h,t}| \end{array}$$

(30) $$\begin{array}{r} MAPE=\frac{1}{N}\overset{N}{\underset{t=1}{\sum }}|\frac{A_{t}-P_{t,h}}{A_{t}}|\ldots \end{array}$$

(31) $$\begin{array}{r} sMAPE=\frac{100\%}{n}\overset{n}{\underset{t=1}{\sum }}\frac{\left|P_{t,h}-A_{t}\right|}{{\left(\left|A_{t}|+|P_{t,h}\right|\right)}^{2}} \end{array}$$

where A_t_ and P_t_ denote actual and predicted values.

These metrics are commonly used techniques to evaluate the accuracy of different point forecasts of the competing models. The most popular and widespread is MAPE as it is very effective, easily understandable, and interpretable. It measures the prediction accuracy as a percentage and can be calculated as the average absolute percent error for each period minus actual values divided by actual values. Subsequently, RMSE, MAE, and sMAPE have also been extensively applied in the literature, although the interpretation of RMSE is more challenging to understand (47).

### Analysis and Discussion

In this section, we discuss different time series model fittings, including the proposed hybrid model, and summarize the main findings of this study. All the COVID-19 data of the four countries were initially arranged into an excel sheet and then for further analysis, RStudio version 1.3.1093 and Python 3.3.6 with Jupyter Notebook were used. The data were first decomposed by successfully implementing the EEMD method into different IMFs, followed by finding out the stationary IMFs using the well-known ADF test. The results of which appear to tally with the authors' expectations that the high-frequency IMFs are mostly stationary and clustered around their mean.

From Tables 1–4, it can be seen that the ADF test results for which predefined value of α = *0.05*, the calculated *p-*value is less than the pre-specified alpha value that leads to the rejection of the null hypothesis that the given IMF is nonstationary, the nonstationary IMFs are not used to build our model, therefore their means are not required. The tabulated results of the ADF test confirm that the majority of the IMFs ranging from 1 to 4 for all the four countries, both for daily confirmed cases and daily deaths, are stationary. These are the most relevant findings and, perhaps, the most significant part of the composition of the proposed hybrid model based on EEMD and ETS approaches. The grand mean given in the tables presents the average short-term variations in the data. These short-term fluctuations are then subtracted from the original signal to get denoised COVID data as an ingredient for the ETS model followed by predictions. The values of smoothing parameters, AIC, BIC, and the type of models are presented in Supplementary Table 4.

At present, based on the minimum values of AIC and BIC, the best candidate ETS model is chosen for prediction, e.g., for Italy's daily confirmed cases, the best-reported model is ETS (M, Ad, M) which means that errors are multiplicative, and the trend in the data is damped additive with multiplicative seasonality. Similarly, to avoid repetition, the same description and interpretation can be made for other models as well.

### Model Comparison

In this section, the proposed hybrid model is evaluated along with different selected time series models that demonstrate prediction results in the case of nonlinear and nonstationary COVID-19 data for the four selected countries. Here, we used a total of 11 methods; of these, 9 are conventional time series, one is a simple neural network with autoregressive terms (NNAR), and one is an RNN with LSTM), and the proposed hybrid method is based on EEMD and ETS models. We also checked the prediction performance of different potential hybrid models, namely, EMD-ARIMA, EMD-ETS, EEMD-ARIMA, and EEMD-ETS, of which the best hybrid model is chosen and the same is then compared with the competing models in terms of performance. To avoid confusion, we reported the best candidate model out of all potential hybrid models. The experimental results of the overall performance of these selected models are presented in terms of the following four measures, i.e., RMSE, MAE, MAPE, and sMAPE.

A key strength of this research lies in the fact that the prediction performance of our proposed model is equally efficient in all scenarios, i.e., for daily confirmed cases and daily deaths for all 4 countries. It can be verified easily from the investigational results presented in Tables 5–12 that the four statistical measures of the suggested model are minimum. The values of RMSE for daily confirmed cases and deaths of Italy are **2404.13** and **77.86**. The second-best model based on the values of RMSE in this competition is ETS with an RMSE value of **2552.25**, and the well-known ARIMA model stands in the third position with an RMSE of **2711.67**. Similarly, the values of MAE, MAPE, and sMAPE of our developed hybrid model are also minimum. Interestingly, the ARIMA model beats the ETS model in these metrics and stands in the second position in this forecast competition of COVID-19 for daily confirmed cases but failed to show good prediction results for daily deaths data (Table 6). In this scenario, the ETS model stands in the second position with minimum values of MAE, MAPE, and sMAPE after the proposed hybrid model.

**Table 5:** Performance of different models for 7 days prediction of Italy's daily confirmed cases.

**Method**	**RMSE**	**MAE**	**MAPE**	**sMAPE**
Mean	31998.353	31613.531	948.659	1.645
SES	5712.565	4365.085	11.54	0.124
Naïve	5712.774	4365.144	11.545	0.126
Theta	3526.853	2665.955	8.457	0.078
TBATS	4091.085	3568.494	9.636	0.107
HW	8790.664	7661.137	18.088	0.206
Damped	8200.137	6999.388	16.837	0.191
ETS	2552.256	2434.029	6.985	0.07
ARIMA	2711.679	2163.304	6.308	0.066
NNAR	4820.24	4019.41	11.667	0.112
LSTM	4874.481	3905.596	11.904	11.503
Hybrid EEMD-ETS	2404.163	1969.82	5.125	0.042

**Table 6:** Performance comparison of different models for 7 days prediction of Italy's daily deaths.

**Method**	**RMSE**	**MAE**	**MAPE**	**sMAPE**
Mean	362.743	343.019	218.311	1.009
SES	139.556	121.623	28.576	0.252
Naïve	129.816	118.714	26.617	0.245
Theta	142.302	123.514	29.296	0.256
TBATS	87.509	75.007	14.687	0.158
HW	106.762	78.143	14.153	0.164
Damped	87.145	74.356	14.47	0.156
ETS	87.144	74.351	14.465	0.153
ARIMA	95.504	81.858	16.348	0.171
NNAR	115.198	105.808	22.691	0.218
LSTM	86.34	75.962	17.058	0.158
Hybrid EEMD-ETS	77.867	70.54	14.049	0.141

**Table 7:** Performance comparison of different models for 7 days prediction of France's confirmed cases.

**Method**	**RMSE**	**MAE**	**MAPE**	**sMAPE**
Mean	37398.196	30671.534	487.759	1.317
SES	27427.455	26493.717	48.953	0.61
Naïve	31616.717	30863.438	51.238	0.675
Theta	27631.862	26714.523	49.135	0.614
TBATS	28348.173	27378.319	49.297	0.622
HW	31772.71	30910.284	52.713	0.677
Damped	27561.259	26395.92	49.811	0.609
ETS	31773.431	30910.951	52.71	0.677
ARIMA	26013.185	24794.244	47.679	0.581
NNAR	29952.856	27340.347	53.578	0.618
LSTM	27989.713	26956.326	49.987	0.618
Hybrid EEMD-ETS	23252.42	14253.33	40.654	0.353

**Table 8:** Performance comparison of different models for 7 days prediction of France's daily deaths.

**Method**	**RMSE**	**MAE**	**MAPE**	**sMAPE**
Mean	539.424	420.586	275.178	0.996
SES	337.894	282.903	50.15	0.486
Naïve	422.35	395.571	47.832	0.626
Theta	281.609	214.945	40.914	0.366
TBATS	298.616	272.944	39.239	0.475
HW	359.555	328.171	44.652	0.55
Damped	328.118	298.227	46.379	0.51
ETS	299.161	265.928	42.413	0.461
ARIMA	288.136	244.529	41.183	0.426
NNAR	146.13	124.111	21.697	0.237
LSTM	335.04	310.608	46.969	0.528
Hybrid EEMD-ETS	102.733	82.378	14.101	0.146

**Table 9:** Performance comparison of different models for 7 days prediction of Germany's confirmed cases.

**Method**	**RMSE**	**MAE**	**MAPE**	**sMAPE**
Mean	16654.38	16243.24	654.767	1.517
SES	5948.078	4715.722	20.153	0.24
Naïve	5948.227	4715.857	20.154	0.24
Theta	1385.131	1000.46	6.46	0.063
TBATS	1372.604	1008.138	6.015	0.529
HW	6933.016	6255.89	25.386	0.302
Damped	6652.431	5843.291	24.053	0.286
ETS	1772.325	1401.285	7.065	1.97
ARIMA	3043.065	2901.103	13.631	0.147
NNAR	2320.36	1904.506	10.035	0.109
LSTM	4593.045	3566.093	16.485	0.191
Hybrid EEMD-ETS	1298.967	935.492	5.348	0.053

**Table 10:** Performance comparison of different models for 7 days prediction of Germany's daily deaths.

**Method**	**RMSE**	**MAE**	**MAPE**	**sMAPE**
Mean	140.659	121.227	279.69	1.024
SES	79.27	72.855	56.041	0.505
Naïve	79.271	72.857	56.043	0.505
Theta	51.394	40.253	27.565	0.228
TBATS	38.225	30.126	20.943	0.23
HW	59.52	48.717	28.418	0.349
Damped	58.292	49.025	30.143	0.35
ETS	37.547	32.468	22.259	0.24
ARIMA	47.14	37.354	26.018	0.258
NNAR	91.139	1117.111	20.697	0.302
LSTM	61.177	51.285	35.68	0.362
Hybrid EEMD-ETS	17.604	15.01	10.258	0.105

**Table 11:** Performance comparison of different models for 7 days prediction of UK's daily cases.

**Method**	**RMSE**	**MAE**	**MAPE**	**sMAPE**
Mean	20472.959	20003.733	451.902	1.373
SES	4459.872	3418.096	14.555	0.136
Naïve	4505.504	3390.143	14.558	0.134
Theta	4165.609	3253.262	14.716	0.133
TBATS	4355.468	3380.606	14.399	0.134
HW	4084.515	3444.378	13.839	0.137
Damped	4184.243	3437.578	14.117	0.137
ETS	3954.365	3333.344	13.418	0.133
ARIMA	4288.603	3226.114	13.809	0.127
NNAR	4686.289	3288.208	14.549	0.13
LSTM	4515.138	3395.429	14.597	0.135
Hybrid EEMD-ETS	4076.516	3130.997	12.573	0.123

**Table 12:** Performance comparison of different models for 7 days prediction of UK's daily deaths.

**Method**	**RMSE**	**MAE**	**MAPE**	**sMAPE**
Mean	271.024	225.884	120.689	0.663
SES	167.729	148.916	41.115	0.409
Naïve	169.672	152.98	42.816	0.418
Theta	119.826	88.672	26.12	0.216
TBATS	114.393	109.527	29.814	0.308
HW	154.058	132.409	29.813	0.364
Damped	151.3	137.454	33.513	0.379
ETS	75.049	68.648	19.067	0.201
ARIMA	73.45	61.904	17.049	0.189
NNAR	95.206	105.765	41.987	0.487
LSTM	154.763	141.574	36.855	0.391
Hybrid EEMD-ETS	70.954	60.976	15.711	0.118

The experimental results for France's daily deaths and daily confirmed cases presented in Tables 7, 8 show that our model outperformed other models in this forecast competition, while the well-known ARIMA model's performance is much better than his strong rival, the ETS model, and stands with the second position with minimum values of RMSE, MAE, and sMAPE.

Investigational results for Germany and the UK are shown in Tables 9–12. It can be verified from the values of four statistical metrics, i.e., RMSE, MAE, MAPE, and sMAPE, that the prediction performance of our suggested model is better than the other conventional and machine learning methods. The ETS model again outperformed the classical ARIMA model and holds the second position for Germany and the UK.

**Figure 5:**
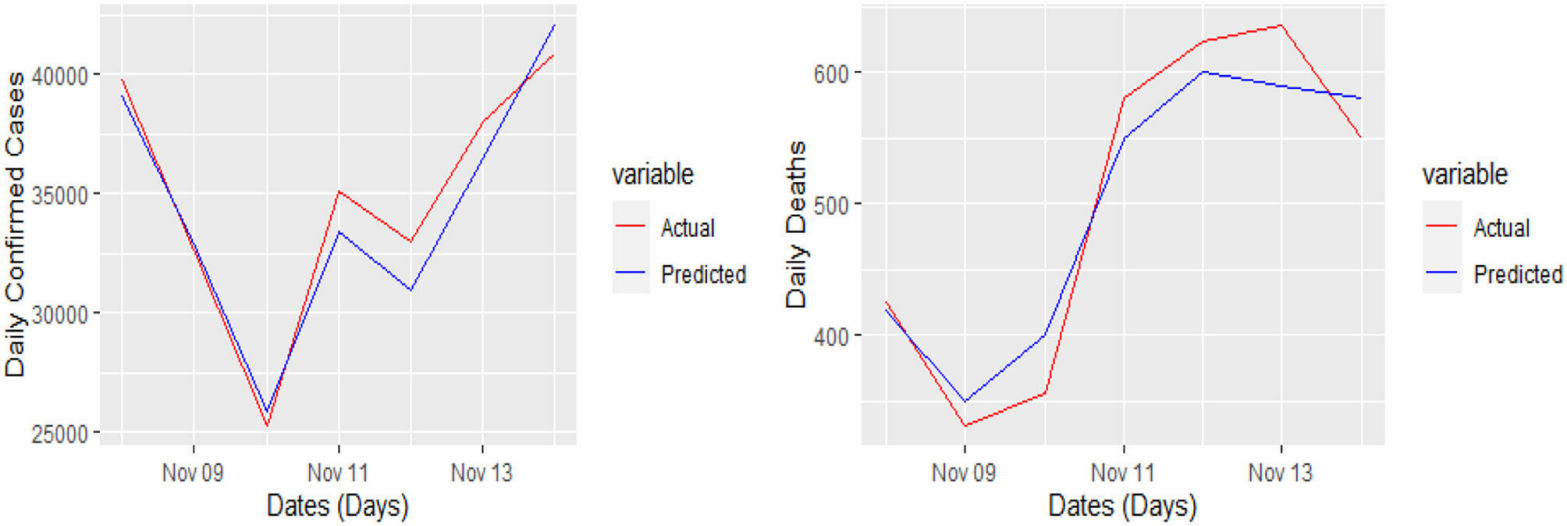
Actual and predicted 7 days daily confirmed cases and deaths for Italy from COVID-19.

**Figure 6:**
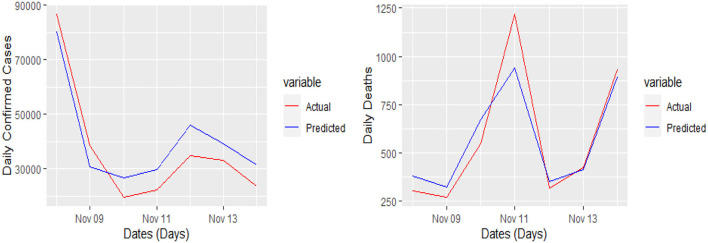
Actual and predicted 7 days daily confirmed cases and daily deaths in France from COVID-19.

**Figure 7:**
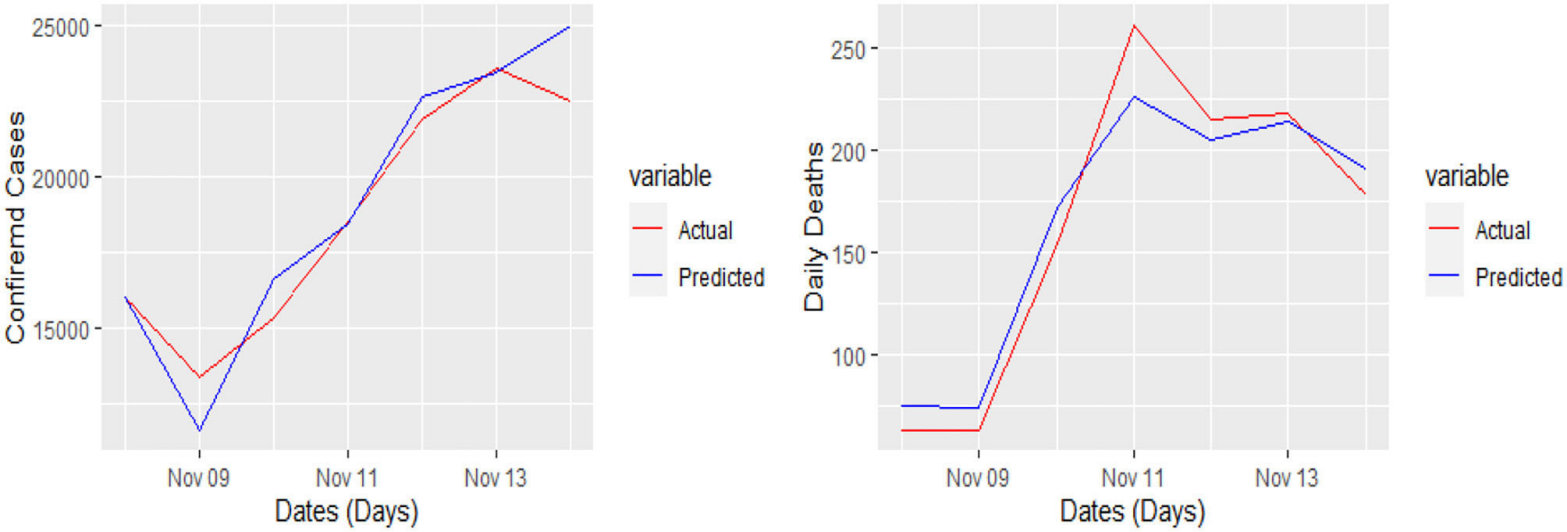
Actual and predicted 7 days daily confirmed cases and daily deaths from COVID-19 in Germany.

**Figure 8:**
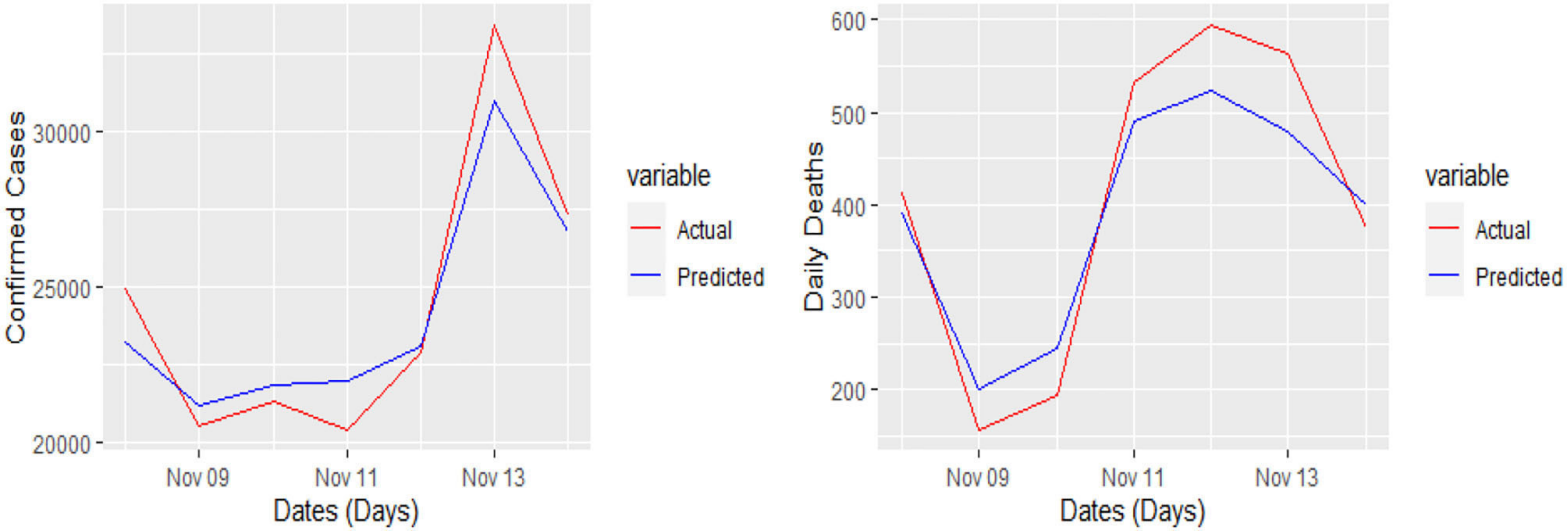
Actual and predicted 7 days daily confirmed cases and daily deaths from COVID-19 in the UK.

The 7 days prediction was made by implementing our proposed model. To save space, we are not reporting these values here; a snapshot will better reflect the scope of our study. To make the prediction clear and understandable, we presented the actual and predicted values schematically through Figures 5–8 for each country and each case. In all these cases, the actual and predicted daily confirmed cases and daily deaths are denoted by solid red and blue lines, respectively.

Yet, the actual and predicted values are far from each other but the direction accuracy of our prediction is more than 90%, which can be verified through Figures 5–8; such a tremendous direction accuracy will help the governments for better policies to stop the spread of the pandemic.

## Conclusion

Prediction of the pandemics is always interesting, and there are numerous areas of research for data practitioners. Accurate prediction of pandemics is of great importance as it will help the governments to implement their resources in a better manner to stop the spread and save the precious lives of their citizens. The main conclusion of this study is drawn together and presented in this section. In most of the previous studies, the researchers used a single mathematical or statistical model to predict the accurate trajectory of the COVID-19 and, therefore, criticized for its poor prediction performance. The key objective of this research work is to propose a novel method to predict the contagious COVID-19 daily confirmed cases and deaths in four major European countries, i.e., Italy, France, Germany, and the UK. A key strength of this research lies in the fact that we proposed a hybrid method that is based on EEMD and univariate time series ETS model. Thus, the suggested technique is very appropriate for prediction with nonlinear and nonstationary data. Our proposed model is not an ensemble model as we did not utilize all the subcomponents after decomposing the COVID-19 data into different IMFs and single monotone residual by implementing the method of EEMD, we used only stationary IMFs to build our model. After successfully implementing the model, we used it for short-term forecasting of only 7 days. A comparison is made with other conventional univariate time series, NNAR and LSTM models. Based on the investigational results of the four statistical metrics, i.e., RMSE, MAE, MAPE, and sMAPE, the proposed model outperformed the other models, indicating that it is a promising tool for COVID-19 prediction. Surprisingly, the univariate single ETS and ARIMA model stands second in this competition and outperformed the NNAR and LSTM model, while we were expecting that the deep neural network LSTM model will perform better than the traditional univariate time series models except the suggested one.

In the future, we are looking to use our proposed algorithm for other countries' COVID-19 data by using different variables, namely, daily recoveries, daily hospitalized patients, and spread rate as well as to check its performance on other univariate time series datasets, namely, stock returns, exchange rates, wind speed, temperature, rainfall, earthquakes, tourist arrival, and crude oil. In short, we are planning to test the accuracy of our proposed model on any nonlinear and nonstationary univariate time series data.

Our study has some drawbacks that require additional investigation. First, as the data are very limited, therefore, the performance of the model will be checked by using it on a longer series and long-term forecasting.

To end with, in this research article, we proposed a hybrid EEMD-ETS model to predict the daily confirmed cases and daily deaths from the current pandemic of COVID-19 using Italy, France, Germany, and UK datasets.

## Data Availability Statement

The datasets presented in this study can be found in online repositories. The names of the repository/repositories and accession number(s) can be found below: The data is already included in the article.

## Author Contributions

DK, AAl, and AAf: conceptualization. DK, HA, and AAf: formal analysis and methodology. HA: funding acquisition. DK, MA, NI, and UK: investigation, resources, and writing–original draft. AAf: project administration. DK and UK: software. DK: supervision. DK, HA, and AAl: validation. MA and NI: visualization. HA, AAl, and AAf: writing–review and editing. All authors contributed to the article and approved the submitted version.

## Funding

This study was funded by Taif University Researchers Supporting Project number (TURSP-2020/279), Taif University, Taif, Saudi Arabia.

## Conflict of Interest

The authors declare that the research was conducted in the absence of any commercial or financial relationships that could be construed as a potential conflict of interest.

## Publisher's Note

All claims expressed in this article are solely those of the authors and do not necessarily represent those of their affiliated organizations, or those of the publisher, the editors and the reviewers. Any product that may be evaluated in this article, or claim that may be made by its manufacturer, is not guaranteed or endorsed by the publisher.
