# Supplementary material for: Short-Term Prediction of COVID-19 Using Novel Hybrid Ensemble Empirical Mode Decomposition and Error Trend Seasonal Model
Source: Front Public Health. 2022 Jul 29;10:922795. doi: 10.3389/fpubh.2022.922795 (PMC9374278; doi:10.3389/fpubh.2022.922795)
Supplement: Supplementary file 1 [file Data_Sheet_1.zip › Figure 2.pdf]

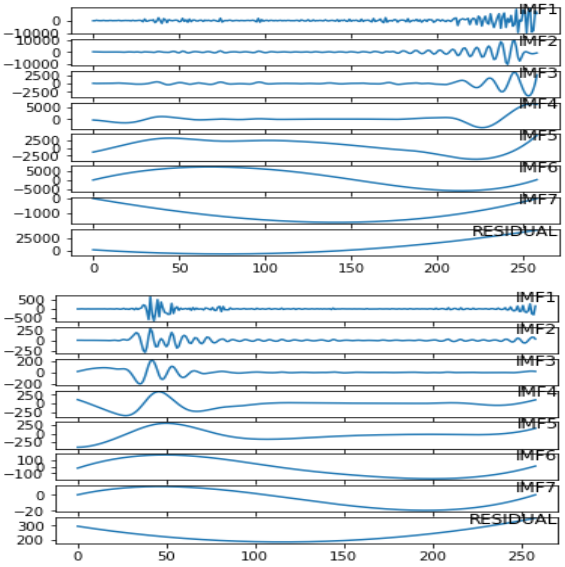

Supplementary Fig. 2. The IMF components of France daily confirmed cases and deaths using the EEMD method
