# Supplementary material for: Short-Term Prediction of COVID-19 Using Novel Hybrid Ensemble Empirical Mode Decomposition and Error Trend Seasonal Model
Source: Front Public Health. 2022 Jul 29;10:922795. doi: 10.3389/fpubh.2022.922795 (PMC9374278; doi:10.3389/fpubh.2022.922795)
Supplement: Supplementary file 1 [file Data_Sheet_1.zip › Table 1.docx]

Supplementary Table 1. Different components of the ETS model

| **Trend Type** | **Seasonal Type** |
| --- | --- |
|  | **N A M** |
| N | NN NA NM |
| A | AN AA AN |
| A_d_ | A_d_N A_d_A A_d_M |
| M | MN MA MM |
| M_d_ | M_d_N M_d_A M_d_M |
