# Supplementary material for: Short-Term Prediction of COVID-19 Using Novel Hybrid Ensemble Empirical Mode Decomposition and Error Trend Seasonal Model
Source: Front Public Health. 2022 Jul 29;10:922795. doi: 10.3389/fpubh.2022.922795 (PMC9374278; doi:10.3389/fpubh.2022.922795)
Supplement: Supplementary file 1 [file Data_Sheet_1.zip › Table 2.docx]

Supplementary Table 2. Descriptive statistics of confirmed cases from COVID-19

| **Country** | **Cumulative Confirmed**  **Cases** | **Average Daily**  **Cases** | **Maximum Daily**  **Cases** | **SD** |
| --- | --- | --- | --- | --- |
| Italy | 1107303 | 4164 | 40902 | 8128 |
| UK | 1317500 | 4953 | 33470 | 7067 |
| Germany | 773556 | 2908 | 23542 | 4715 |
| France | 1886286 | 7096 | 86794 | 12806 |
