# Supplementary material for: Short-Term Prediction of COVID-19 Using Novel Hybrid Ensemble Empirical Mode Decomposition and Error Trend Seasonal Model
Source: Front Public Health. 2022 Jul 29;10:922795. doi: 10.3389/fpubh.2022.922795 (PMC9374278; doi:10.3389/fpubh.2022.922795)
Supplement: Supplementary file 1 [file Data_Sheet_1.zip › Table 3.docx]

Supplementary Table 3. Descriptive statistics of deaths from COVID-19

| **Country** | **Cumulative Deaths** | **Average Daily**  **Deaths** | **Maximum Daily**  **Deaths** | **SD** |
| --- | --- | --- | --- | --- |
| Italy | 44139 | 166 | 971 | 225 |
| UK | 51304 | 193 | 1224 | 276 |
| Germany | 12378 | 47 | 315 | 69 |
| France | 43559 | 164 | 2003 | 277 |
