# Supplementary material for: Short-Term Prediction of COVID-19 Using Novel Hybrid Ensemble Empirical Mode Decomposition and Error Trend Seasonal Model
Source: Front Public Health. 2022 Jul 29;10:922795. doi: 10.3389/fpubh.2022.922795 (PMC9374278; doi:10.3389/fpubh.2022.922795)
Supplement: Supplementary file 1 [file Data_Sheet_1.zip › Table 4.docx]

Supplementary Table 4. Summary of the proposed hybrid EEMD-ETS model

| **Country** | **Signal** | **Smoothing Parameters** | **AIC** | **BIC** | **Model Type** |
| --- | --- | --- | --- | --- | --- |
| Italy | Confirmed Cases | *α=0.194, β=0.0563*  γ = 0.2101, π=0.9366 | 4466.130 | 4512.369 | ETS(M,Ad,M) |
|  | Deaths | *α=0.5376, β=0.0001*  γ = 0.0001 | 3405.359 | 3448.041 | ETS (M, A, M) |
| France | Confirmed Cases | *α=0.1072, β=0.0226*  γ = 0.5269 | 5584.606 | 5627.288 | ETS (A, A, A) |
|  | Deaths | *α=0.1261, β=0.049*  γ = 0.4158, π=0.0.9328 | 3231.744 | 3277.982 | ETS(M,Ad,M) |
| Germany | Confirmed Cases | *α=0.2976, β=0.0979*  γ = 0.2248 | 4465.449 | 4508.131 | ETS (M, A, M) |
|  | Deaths | *α=0.1508, β=0.0507*  γ = 0.0619 | 2644.842 | 2687.524 | ETS (M, A, M) |
| UK | Confirmed Cases | *α=0.4276, β=0.0461*  γ = 0.1387 | 4606.031 | 4648.713 | ETS (M, A, M) |
|  | Deaths | *α=0.193, β=0.0683*  γ = 0.0463, π=0.9422 | 3164.785 | 3211.024 | ETS(M,Ad,M) |
